# Prevalence of social anxiety disorder and symptoms among Chinese children, adolescents and young adults: A systematic review and meta-analysis

**DOI:** 10.3389/fpsyg.2022.792356

**Published:** 2022-08-22

**Authors:** Xinfeng Tang, Qiwen Liu, Fangtong Cai, Hui Tian, Xincheng Shi, Suqin Tang

**Affiliations:** ^1^Department of Psychology, Renmin University of China, Beijing, China; ^2^Department of Sociology, Law School, Shenzhen University, Shenzhen, China; ^3^School of Psychology, Shenzhen University, Shenzhen, China

**Keywords:** children, adolescents, young adults, China, prevalence, social anxiety

## Abstract

The objective of the study is to provide a reliable estimate of the pooled prevalence of social anxiety disorder (SAD) and social anxiety symptoms (SAS) among children, adolescents, and young adults (CAYA) in China. Meta-analysis is used to provide pooled-prevalence rate of SAD and SAS. Literature searches were conducted in both English and Chinese databases from the database's inception to April 2019. Eleven studies were identified for SAD, and 17 were included for SAS. The results revealed a pooled prevalence of SAD of 2.1% (95% CI: 1.2–3.8%) with high between-studies heterogeneity (*Q* = 1,055.2, *I*^2^ = 99.1%, *p* < 0.001). The pooled prevalence estimate of SAS was 23.5% (95% CI: 18.6–29.3%), also with significant heterogeneity (*Q* = 1,019.3, *I*^2^ = 98.4%, *p* < 0.001). Different diagnostic tools or self-report scales reported significant different prevalence of SAD or SAS. Further analysis stratified by gender, age, sampling methods, economic status, and risk of bias were performed. Limitations include the high level of heterogeneity between studies, inadequate number of the studies, and significant differences in prevalence caused by measurements.

**Systematic review registration:**

https://www.crd.york.ac.uk/prospero/display_record.php?ID=CRD42020149591, identifier: PROSPERO CRD42020149591.

## Introduction

Social anxiety disorder (SAD) is quite common among young people, with a lifetime prevalence rate ranging from 3.5 to 9.1% aged 10-24 in western countries such as the United State, Germany and Austria (Stein et al., 2001; Burstein et al., 2011; Knappe et al., 2011; Wagner et al., 2017). The core features of SAD are excessive fear of scrutiny by others and avoidance of social situations in which embarrassment or humiliation might occur (American Psychiatric Association., 2013). Social anxiety brings a series of problems such as impaired academic and global functioning (Ranta et al., 2009; Edlund et al., 2018; Finsaas et al., 2020), loneliness (Bernstein et al., 2008; Eres et al., 2021), low level of social support from classmates (Coyle and Malecki, 2018), poor social skills (Scharfstein et al., 2011; Lau et al., 2022), and high rates of sickness absence and unemployment (Amin et al., 2019). The onset of SAD is early, with a median age of 13 years in epidemiological studies (Kessler et al., 2005), and there are few new cases after 25 years of age (Wittchen and Fehm, 2003; Stein et al., 2017). In clinical samples, SAD is the most chronic anxiety disorder, with a 37–56% remission rate (Bruce et al., 2005; Springer et al., 2018).

Due to its significant negative consequences, SAD has received much research and clinical attention. However, significant social anxiety symptoms (SAS) should not be ignored because social anxiety is on a continuous spectrum from absence of social fear through normal anxiety and shyness to SAD (McNeil, 2001). Studies have shown that even subthreshold social anxiety (≥one social phobia symptoms in DSM-IV plus avoidance) brings significant psychosocial impairment and elevated rates of other psychiatric disorders (Crum and Pratt, 2001; Merikangas et al., 2002; Filho et al., 2010). In the present study, SAD refers to a type of an anxiety disorder that meet the DSM-5 or ICD-11 diagnosis. SAS, on the other hand, refers to the symptoms and manifestations of social anxiety which is measured by valid self-report scales.

Epidemiological surveys revealed significant cultural differences in the prevalence of social anxiety symptoms and disorder. A review shows that the prevalence of SAD among adults in Asian countries is significantly lower than that in Europe and the United States (Hofmann et al., 2010). For example, Asian countries such as China, South Korea, and Japan had 12-month prevalence rates of 0.2% (Shen et al., 2006), 0.2% (Cho et al., 2007), and 0.8% (Kawakami et al., 2005), respectively. Meanwhile, the prevalence of SAD in some European and American countries such as the United States, the Netherlands, and Australia ranged between 4.2 and 7.1% (Bijl et al., 1998; Ruscio et al., 2008; Crome et al., 2015). Interestingly, the phenomenon is reversed in self-report measurement. A meta-analysis found that individuals of Asian heritage had higher self-reported social anxiety scores than those of European heritage in 28 out of 32 independent studies and yielded a mean effect size of *d* = 0.36 (Krieg and Xu, 2015). One possible reason for this difference is that Asian individuals belong to collectivism and are more likely to feel embarrassment and anxiety in social interactions, and therefore have higher social anxiety symptoms. However, Asian culture acquiesces or even appreciates social shyness and anxiety. Individuals with high social anxiety symptoms do not necessarily bring about impairment in social functioning in Asian societies, and therefore exhibit lower prevalence of social anxiety disorder (Heinrichs et al., 2006).

It is not clear whether a similar phenomenon exists in children and adolescent populations. There is a lack of large-scale epidemiological surveys or meta-analyses to report the prevalence of SAD or SAS among Chinese children, adolescents, and young adults (CAYA). Some studies found that the point prevalence of SAD in primary and secondary schools was as low as 0.6% (Ye et al., 2013), while others reported a prevalence rate of 2.7% (Su et al., 2006). The findings of SAS prevalence were also inconsistent. One previous study found that 6.9% of Chinese primary school students experienced severe social anxiety symptoms as measured by the Social Anxiety Scale for Children (SASC; Cai, 1998). However, with the same assessment tool and cutoff point, one more recent study reported a rate of 26.3% in a similar population (Gao et al., 2013). These inconsistencies might be due to the use of different age groups of CAYA, sampling frames, sampling methods, and other factors.

Compared with depression, PTSD, and other mental health issues, people have less knowledge and poorer recognition of social anxiety (Katzelnick et al., 2001; Coles et al., 2016). The public's underestimate of the severity of social anxiety could be due to the inaccurate estimates of the prevalence of social anxiety, which might further hinder the clinicians' detection and recognition of social anxiety and provide treatment to people who are in need.

Therefore, a meta-analysis focusing on the prevalence of social anxiety among Chinese CAYA is warranted. The purpose of the current study was to combine the prevalence rates in existing surveys to provide a more reliable prevalence estimate of SAD and its symptoms in Chinese CAYA. For social anxiety disorder, the prevalence refers to the proportion of individuals who are positive on a certain diagnostic tool. For social anxiety symptoms, the prevalence is the proportion of individuals who exceed a cutoff on a self-rated scale. The most appropriate test cutoff value is determined with a compromise between sensitivity and specificity based on receiver operating characteristic (ROC) analysis (Habibzadeh et al., 2016). Although there are some drawbacks to using a dichotomous approach for a psychological disorder which lies on a continuum, this approach is simple to understand and can be of great value to the public and to clinical practice, especially in China where relevant data are lacking.

## Materials and methods

We followed the Preferred Reporting Items for Systematic Reviews and Meta-Analyses (PRISMA; Liberati et al., 2009) and Meta-Analysis of Observational Studies in Epidemiology (MOOSE; Stroup et al., 2000) guidelines throughout this meta-analysis. This review has been registered at PROSPERO (Registration Number: CRD42020149591).

### Search strategy

We searched the following online English-language databases: MEDLINE, PsycINFO, EMBASE, and Web of Science. Chinese databases were also searched including the China National Knowledge Infrastructure Database (CNKI), the Wanfang database and the Chinese Scientific Journal Database (VIP Database). Reference lists of relevant review articles and all included articles, as well as papers citing these relevant studies in Google Scholar or CNKI, were searched by hand for additional studies. The review examined all journal articles published until July 2020. The search was restricted to English and Chinese language articles. The search terms can be seen in Supplementary material 1.

### Inclusion and exclusion criteria

The following inclusion criteria were applied: (1) CAYA aged 6–25; (2) original studies that reported the prevalence rate of SAD or symptoms; and (3) the use of a standardized assessment procedure deriving diagnosis of SAD or valid measures with good psychometric properties to assess social anxiety symptoms (Leffler et al., 2015; Wong et al., 2016; Hunsley and Mash, 2018); diagnostic and self-report instruments are listed in Supplementary material 2; (4) Most studies only reported point prevalence of SAD in Chinese CAYA. Therefore, we only included studies which reported point prevalence of SAD in the current meta-analysis. (5) For SAS, the scales in the included studies must contain a definite cutoff which is supported by empirial research.

The following studies were excluded from this meta-analysis: (1) reviews, case reports, comments, letters, or editorials; (2) studies that only focused on a specific population such as CAYA who were obese, bullied, or experienced physical (e.g., stuttering) or mental illness (e.g., depression); and (3) articles that could not be retrieved in full-text form through online databases, library requests, or email correspondence with the authors of the studies.

### Data extraction and coding

Two reviewers (FC and QL) screened the title and/or abstracts independently and then retrieved the full texts and independently assessed them based on the inclusion and exclusion criteria. They used a standardized form to extract information such as authors, publication year, participants and setting, assessment tools, sample size, prevalence rate, and other subgroup data (e.g., gender). If the study was carried out at multiple time points, then data from the first time point were used because there might have been missing participants in the follow-ups. If multiple studies were based on the same dataset, the study with a larger sample size was included. Interrater reliability was calculated for continuous variables (e.g., prevalence, sample size) using intraclass correlation coefficients (ICC) and for categorical moderators (e.g., gender, sampling method) using Cohen's kappa. Interrater reliability was high (ICC = 0.99 and κ = 0.87 for continuous and categorical variables, respectively), and discrepancies were resolved through discussion. If a consensus could not be reached, other reviewers (ST and XT) discussed until they reached a consensus. Missing or additional data were requested from the original authors.

### Quality assessment

We assessed the quality of each study according to the Risk of Bias Tool for Prevalence Studies developed by Hoy et al. (2012). The tool has 10 items including external validity and internal validity subscales. The external validity subscale has four items including representation of the national population, sampling frame, random sampling, and nonresponse bias. The internal validity subscale has six items: data collected directly from the participants or a proxy, case definition, quality of instruments, consistency of data collection mode, duration of the prevalence period, and calculation of prevalence (see Supplementary material 3). Each item was assigned a score of 1 (low risk of bias) or 0 (high risk of bias). The sum of these items was the total score ranging from 1–10. Consistent with previous studies (Aminde et al., 2016), each study was classified as having a low (≥ 8), moderate (6–7), or high (≤5) risk of bias. Two investigators (XS and HT) independently rated the included studies, and inconsistencies were resolved by consensus or by the decision of other authors (ST and XT).

### Meta-analytic procedures

Due to the between-studies heterogeneity in the current review, a random effects model was used to combine prevalence estimates from multiple studies (Borenstein et al., 2010). It gave an overall estimate of prevalence rates across studies weighted by sample size. The *I*^2^ statistic was chosen as an indicator of heterogeneity. *I*^2^ values of 25, 50, and 75% are generally interpreted as mild, moderate, and high degrees of heterogeneity, respectively. An *I*^2^ <50% is considered acceptable (Higgins et al., 2003).

We performed subgroup analyses stratified by scales, gender, age group, sampling method, sample size, economic status, risk of bias, etc. Publication bias was assessed using funnel plots and Egger's tests (Egger et al., 1997; Sterne and Egger, 2001). We also calculated the “fail-safe N” to evaluate how many additional studies with zero effect would be needed to nullify the overall effect size. A fail-safe N greater than or equal to five times the number of observations plus 10 indicates a robust result (Rosenthal, 1979). Comprehensive Meta-Analysis (V2.0, Biostat, Englewood, NJ, USA) was used to perform the meta-analyses.

## Results

### Characteristics of included studies

**Figure 1:**
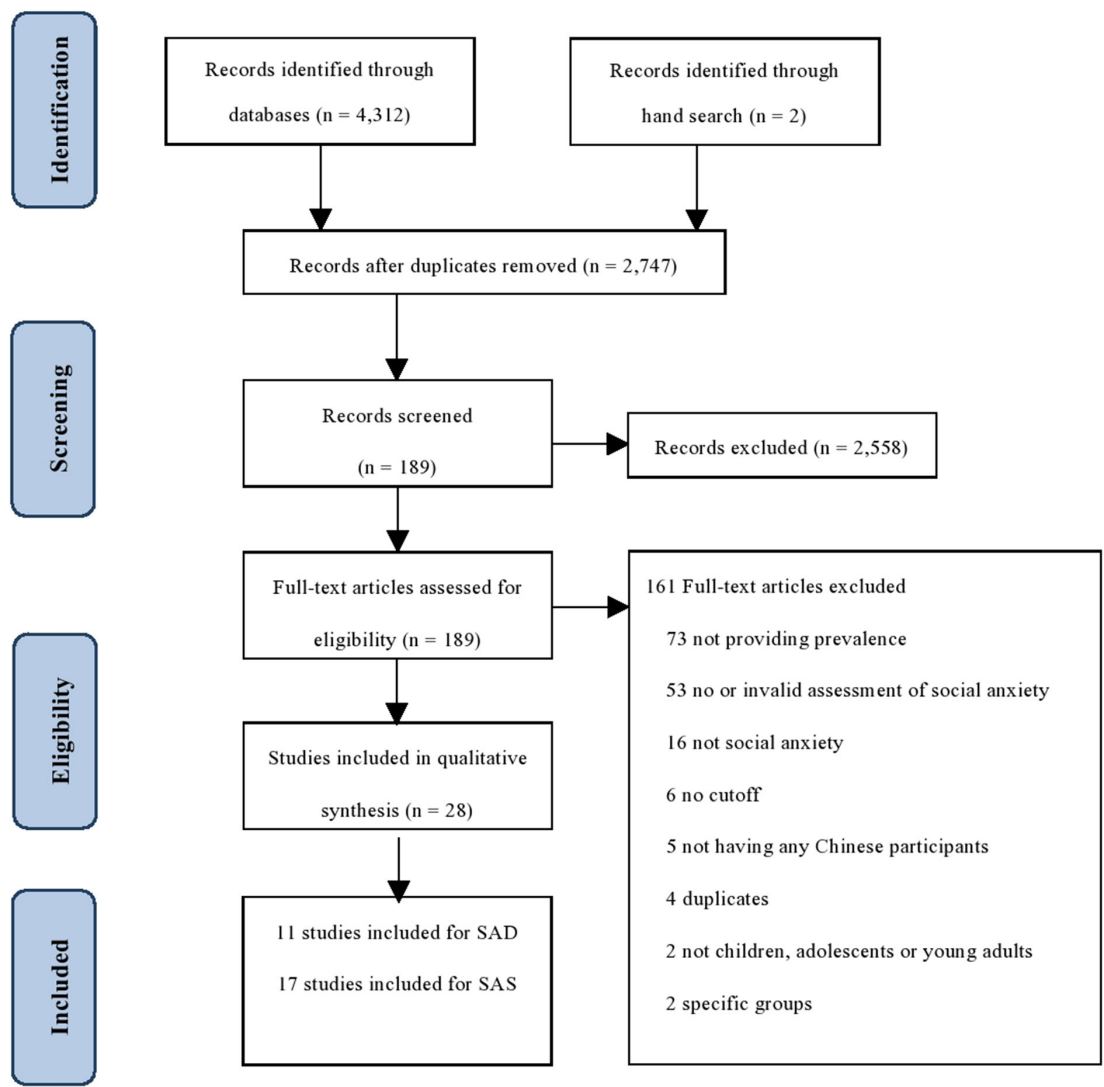
PRISMA flowchart of study selection.

Figure 1 depicts the screening process. According to the search terms, a total of 2,747 unique records were found. Among these, 2,558 were excluded by screening titles and abstracts. A total of 189 full texts were retrieved for further assessment of eligibility. After the full-text screening, 17 articles met the inclusion criteria for social anxiety symptoms, and 11 were included for SAD (the full list of articles is shown in Supplementary material 4). Of the 161 articles that were excluded, 73 did not provide prevalence, 53 did not use valid measures for social anxiety, 16 did not focused on social anxiety, six did not provide a cutoff, five studies were not Chinese participants, and the rest were excluded due to duplicates (*n* = 4) not focusing on CAYA (*n* = 2), or focusing on specific groups (*n* = 1).

Table 1 shows the characteristics of the included studies on SAD. Eleven independent studies were eligible for the meta-analysis, including a total of 60,921 participants. Six diagnostic instruments were used to identify SAD: the Structured Clinical Interview for DSM-IV (SCID-IV; *n* = 3), the Kiddie Schedule for Affective Disorders and Schizophrenia (K-SADS; *n* = 2), the Mini International Neuropsychiatric Interview for Children and Adolescents (MINI-KID; *n* = 2), the Chinese Classification of Mental Disorders Version 3 (CCMD-3; *n* = 2), the Development and Wellbeing Assessment (DAWBA; *n* = 1), and the Composite International Diagnostic Interview Version 3.0 (CIDI 3.0; *n* = 1).

**Table 1:** Social anxiety disorder: study characteristics of the included studies.

**Author (s) (year)**	**Province**	**Diagnostic tools**	**Population**	**Sampling methods**	**Mean age**	**Female percentage**	***N***	**Point prevalence**	**Gender subgroup**	**Risk of bias**
Du et al. (2010)	Sichuan	DAWBA	P3–P6	Probability	10.1	/	1,441	2.6%	/	Low
Guan et al. (2010)	Hunan	K-SADS-PL	P1–J3	Probability	/	45.8%	9,495	1.20%	/	Low
Luan et al. (2014)	Heilongjiang	SCID	U1–U3	Probability	21	53.6%	1,878	8.9%	/	Low
Qu et al. (2015)	Sichuan	MINI-KID	P1–S1	Probability	/	49.6%	19,711	0.70%	/	Low
Su et al. (2003)	Hunan	CCMD-3	P2–P6	Convenience	10.09	48.7%	565	2.5%	Male: 2.4%; Female: 2.5%	Low
Su et al. (2006)	Guangdong	CCMD-3	P1–P6	Probability	9.64	39.0%	2,409	2.7%	Male: 2.6%; Female: 2.9%	Low
Wang et al. (2014)	Seven military regions in China	CIDI 3.0	Military soldiers	Probability	22	2.0%	11,527	3.10%	/	Low
Xiao et al. (2006)	Sichuan	SCID	J1–U4	Probability	16.86	48.0%	2,279	8.2%	Male: 7.62%; Female: 8.35%	Low
Ye et al. (2013)	Hunan	K-SADS and DSM-4	P1–S3	Probability	10.53	47.5%	2,561	0.6%	/	Low
Zhang and Liu (2011)	Shandong	SCID-I/P	J1–U4	Probability	/	48.2%	2,479	7.2%	Male: 7%; Female: 7.4%	Moderate
Zhang et al. (2018)	Jiangsu	MINI-KID	P1–S3	Probability	/	51.7%	6,576	0.2%	/	Low

*DAWBA, The Development and Well-Being Assessment; K-SADS, The Kiddie Schedule for Affective Disorders and Schizophrenia; K-SADS-PL, The Schedule for Affective Disorders and Schizophrenia for School-Age Children-Present and Lifetime version; SCID, Structured Clinical Interview for DSM Disorders; SCID-I/P, The SCID Patient Edition; MINI-KID, The Mini-International Neuropsychiatric Interview for Children and Adolescents; CCMD-3, The Chinese Classification of Mental Disorders Version 3; CIDI 3.0, The Composite International Diagnostic Interview Version 3.0; DSM-4, Diagnostic and Statistical Manual of Mental Disorders (Fourth Edition)*.

*P1–P6, first-year to sixth-year students in junior secondary school; J1–J3, first-year to third-year students in junior secondary school; S1–S3, first-year to third-year students in senior secondary school; U1–U4, first-year to fourth-year students in university*.

The characteristics of studies on SAS is presented in Table 2. Seventeen studies were included involving a total of 17,677 participants. These studies had sample sizes ranging from 137 to 5,162 and covered nine provinces in China. Of the 17 included studies, 14 studies used convenience sampling, and only 3 studies used probability sampling. All studies were conducted in school settings, from primary schools to universities. Three self-report measures with different cutoffs were used to screen SAS: the Social Anxiety Scale for Children (SASC; cutoff ≥ 8, *n* = 5; cutoff ≥ 10, *n* = 6), the Liebowitz Social Anxiety Scale-Self Report (LSAS-SR; cutoff ≥ 38, *n* = 4), and the Social Phobia Inventory (SPIN; cutoff ≥ 20, *n* = 1; cutoff ≥ 25, *n* = 1).

**Table 2:** Social anxiety symptoms: study characteristics of the included studies.

**Author (s) (year)**	**Province**	**Population**	**Sampling methods**	**Mean age**	**Female percentage**	***N***	**Screening tools**	**Cutoff**	**Prevalence**	**Gender subgroup**	**Risk of bias**
Cai (1998)	/	P4–P6	Convenience	/	49.9%	1,030	SASC	≥10	6.9%	Male: 5.2%; Female: 8.2%	Moderate
Chen et al. (2016)	Heilongjiang	P1–P5	Convenience	/	56.2%	539	SASC	≥8	29.3%	Male: 28.8%; Female: 29.7%	Moderate
Cheng et al. (2017)	Taiwan	U1	Convenience	17.4	35.2%	5,162	SPIN	≥25	23.7%	Male: 24.2%; Female: 22.6%	Moderate
Gao et al. (2013)	Heilongjiang	P3–P5	Probability	/	52.3%	927	SASC	≥10	26.3%	Male: 26.5%; Female: 26%	Low
Li et al. (2011)	Jilin	U1–U4	Convenience	/	49.3%	1,362	LASA-SR	≥38	47.6%	/	Moderate
Li et al. (2015)	Zhejiang	U2–U4	Convenience	20.19	54.6%	1,534	LSAS-SR	≥38	41.6%	/	Moderate
Lin et al. (2018)	Xinjiang	P4–P6	Probability	10	48.2%	919	SASC	≥8	28.5%	Male: 26.5%; Female: 30.7%	Low
Qiu and Kang (2014)	/	P4	Convenience	/	48.9%	137	SASC	≥8	26.3%	Male: 22.9%; Female: 29.9%	Low
Shang et al. (2012)	Heilongjiang	P5, P7, J1	Convenience	/	55.1%	1,526	SASC	≥10	13.6%	Male: 12%; Female: 14.6%	Moderate
Su et al. (2015)	Heilongjiang	P3–P5	Probability	/	/	1,393	SASC	≥8	23.5%	Male: 20.2%; Female: 26.3%	Low
Wang (2010)	Anhui	U1–U4	Convenience	/	53.2%	357	SPIN	≥20	8.1%	Male: 6.6%; Female: 9.4%	Low
Wang et al. (2006)	Anhui	P3–P6	Convenience	10.53	46.6%	884	SASC	≥10	14.7%	Male: 12.9%; Female: 16.7%	Low
Wei and Huo (2009)	Beijing	U2–U3	Convenience	20.17	59.6%	324	LSAS-SR	≥38	30.6%	/	Moderate
Wei et al. (2011)	/	U1–U4	Convenience	20.79	90.2%	164	LSAS-SR	≥38	40.2%	/	Low
Wu et al. (2016)	Anhui	P3, P4, J1	Convenience	11.2	48.3%	816	SASC	≥10	15.7%	Male: 16.4%; Female: 15%	Moderate
Yang and Xu (2003)	Jiangsu	P2, P4, P6	Convenience	/	/	303	SASC	≥10	15.8%	/	Moderate
Yu et al. (2015)	Shandong	P5, P6	Convenience	11	46.3%	300	SASC	≥8	43.3%	Male: 49.1%; Female: 36.7%	Moderate

*SASC, Social Anxiety Scale for Children; LSAS-SR, Liebowitz Social Anxiety Scale-self report; SPIN, Social Phobia Inventory*.

*P1–P6, first-year to sixth-year students in junior secondary school; J1–J3, first-year to third-year students in junior secondary school; S1–S3, first-year to third-year students in senior secondary school; U1–U4, first-year to fourth-year students in university*.

### Prevalence of social anxiety among Chinese CAYA

The overall point prevalence of SAD among Chinese CAYA was 2.1% (95% CI: 1.2–3.8%) with significant heterogeneity (*Q* = 1,055.2, *I*^2^ = 99.1%, *p* < 0.001; see Supplementary material 5). The overall pooled prevalence estimates of SAS yielded a crude summary prevalence of 23.5% (95% CI: 18.6–29.3%) with significant heterogeneity present (*Q* = 1019.3, *I*^2^ = 98.4%, *p* < 0.001; see Supplementary material 6).

### Subgroup analysis

The results of subgroup analysis of SAD are presented in Table 3. There were substantial differences in prevalence of SAD between different diagnostic instruments. Studies that employed SCID to identify SAD had the highest prevalence (8.1%; 95% CI: 6.1–10.6%) compared with studies that used CCMD-3 (2.6%; 95% CI: 1.7–4.1%), K-SADS (0.9%; 95% CI: 0.6–1.4%), and MINI-KID (0.4%; 95% CI: 0.3–0.7%; *p* < 0.001). CAYA above 15 years old (6.4%; 95% CI: 3.7–10.9%) had a higher prevalence of SAD than those below 15 years old (1.8%; 95% CI: 1–3.4%; *p* = 0.003). There were no significant differences in prevalence estimates between different economic statuses or genders.

**Table 3:** Pooled event rates of social anxiety disorder grouped by moderators.

	**No. of study**	**Total *N***	**Event rate (95% CI)**	**Heterogeneity**			**Egger's test, *p***
				**Q**	***p***	***I^2^***	
**Diagnostic instruments**							
MINI-KID	2	26,287	0.004 (0.003–0.007)	20.2	0.001	95.1	/
K-SADS	2	12,056	0.009 (0.006–0.014)	6.8	0.01	85.3	/
CCMD-3	2	2,974	0.026 (0.017–0.041)	0.1	0.75	0	/
SCID	3	6,636	0.081 (0.061–0.106)	4.3	0.12	53.7	0.61
*Between-groups*				144.3	0.001		
**Age**							
6–15	4	6,976	0.018 (0.01–0.034)	166.4	0.001	90.4	0.35
15–25	4	18,163	0.064 (0.037–0.109)	211.9	0.001	98.6	0.01
*Between-groups*				8.8	0.003		
**Gender**							
Boy	4	4,231	0.046 (0.027–0.076)	42.2	0.001	92.9	0.28
Girl	4	3,501	0.050 (0.029–0.082)	33.1	0.001	90.9	0.15
*Between-groups*				0.05	0.82		
**Economic status**							
Developed province	3	11,464	0.016 (0.004–0.066)	183.5	0.001	98.9	0.06
Developing province	7	49,457	0.022 (0.009–0.056)	834.1	0.001	99.3	0.53
*Between-groups*				0.139	0.71		

*K-SADS, The Kiddie Schedule for Affective Disorders and Schizophrenia; SCID, Structured Clinical Interview for DSM Disorders; MINI-KID, The Mini-International Neuropsychiatric Interview for Children and Adolescents; CCMD-3, The Chinese Classification of Mental Disorders Version 3*.

Table 4 shows the subgroup analysis of SAS studies. A significant difference in the prevalence of SAS was also observed between different self-reports with different cutoffs. The pooled prevalence was the highest with LSAS-SR with a cutoff of 38 (40%; 95% CI: 31.4–49.3%), followed by SASC with a cutoff of 8 (29.8%; 95% CI: 23.2%−37.3%) and SASC with a cutoff of 10 (14.7%; 95% CI: 11.2–19%; *p* < 0.001). When stratified by the economic status of location where the studies were conducted, participants from developed areas had higher combined prevalence estimates (32.8%; 95% CI: 21.4–42.8%) than those from developing areas (18.9%; 95% CI: 13.9–25.3%). CAYA aged 15–25 had a slightly higher prevalence of SAS (29.8%; 95% CI, 20.9–40.6%) than those aged 6–15 (20.6%; 95% CI, 15.4–26.9%). However, the difference was not significant (*p* = 0.100). Gender, sampling methods, and risk of bias were not significant moderators.

**Table 4:** Pooled event rates of social anxiety symptoms grouped by moderators.

	**No. of study**	**Total *N***	**Event rate (95% CI)**	**Heterogeneity**			**Egger's test, *p***
				**Q**	***p***	***I^2^***	
**Scales**							
**SASC**							
≥8	5	3,288	0.298 (0.232–0.373)	48.2	0.001	91.7	0.38
≥10	6	5,486	0.147 (0.112–0.190)	137.5	0.001	96.4	0.32
**LSAS-SR**							
≥38	4	3,384	0.40 (0.314–0.493)	33.3	0.001	91	0.39
*Between-groups*				32.8	0.001		
**Age**							
6–15	11	8,774	0.206 (0.154–0.269)	354.1	0.001	97.2	0.66
15–25	6	8,903	0.298 (0.209–0.406)	450.8	0.001	98.9	0.85
*Between-groups*				2.7	0.10		
**Gender**							
Boy	12	7,306	0.189 (0.148–0.238)	243.4	0.001	95.5	0.25
Girl	12	5,980	0.207 (0.163–0.259)	168.8	0.001	93.5	0.59
*Between-groups*				0.3	0.59		
**Sampling methods**							
Probability sampling	3	3,239	0.26 (0.145–0.423)	7.5	0.02	73.1	0.33
Convenience sampling	14	14,438	0.23 (0.175–0.296)	1,009.8	0.001	98.7	0.38
*Between-groups*				0.16	0.69		
**Economic status**							
Developed areas	6	8,985	0.328 (0.241–0.428)	423.6	0.001	98.8	0.64
Developing areas	8	7,361	0.189 (0.139–0.253)	189.7	0.001	96.3	0.21
*Between-groups*				6.5	0.011		
**Risk of bias**							
Low	7	4,781	0.224 (0.151–0.318)	119.7	0.001	95	0.64
Moderate	10	12,896	0.244 (0.178–0.324)	871.7	0.001	99	0.56
*Between-groups*				0.12	0.73		

*SASC, Social Anxiety Scale for Children; LSAS-SR, Liebowitz Social Anxiety Scale-self report*.

### Quality assessment and publication bias

For SAD, the majority (10 of 11) of the included studies were assessed as having low risk of bias, and only one study had a moderate risk of bias. The funnel plot (see Supplementary material 7) or Egger test (*p* = 0.28) did not find significant publication bias. The fail-safe N analysis suggested that an additional number of 5,182 studies with non-significant results would be required to reduce the overall effect size to a trivial level at *p* = 0.05, which indicated that the result was robust.

For SAS, seven of 17 studies were rated as having low risk of bias, while the other 10 studies had a moderate risk of bias. Publication bias in the prevalence of SAS was not detected in the funnel plot (see Supplementary material 8) or Egger test (*p* = 0.340). The fail-safe N calculation revealed that 2,627 additional studies with non-significant results would be needed to substantially change the overall effect to a trivial level at *p* = 0.05.

## Discussion

To our knowledge, this review is the first meta-analysis that provides prevalence estimates of SAD and its symptoms in Chinese CAYA.

The review showed that the point prevalence of SAD among Chinese CAYA was 2.1%. Direct comparisons are difficult to conduct because most studies in other countries tend to report 12-month or lifetime prevalence (Burstein et al., 2011; Knappe et al., 2011; Jefferies and Ungar, 2020; Mohammadi et al., 2020). However, there are some studies providing point prevalence rates in countries such as Spain (3.4%; Canals et al., 2019) and Austria (3.5%; Wagner et al., 2017), which had a higher prevalence than Chinese CAYA. This observation is consistent with Hofmann's review, which found that the prevalence of SAD is lower in Asian cultures than in European cultures (Hofmann et al., 2010). Meanwhile, the current study showed that the proportion of Chinese CAYA experiencing SAS was 23.5%. This prevalence estimate is comparable to the prevalence of CAYA in many other countries such as the United Kingdom (28.5%; Fahy et al., 2016), Italy (23.5%; Di Blasi et al., 2015), Spain (25.8%; Calvete, 2014), India (22.9%; Yuvaraj et al., 2018), and Saudi Arabia (25.8%; Hakami et al., 2017).

Our review was partially consistent with the findings of Krieg and Xu (2015)—that is, Chinese CAYA have a lower prevalence of SAD than their western counterparts, however, the prevalence of SAS is similar to their western counterparts.

The similar prevalence of SAS may be due to the fact that Chinese and Western CAYA are exposed to the same risk factors, such as low self-acceptance, negative self-evaluation, and fear of negative evaluation (Li et al., 2019; Peng et al., 2019; Liu et al., 2020). On the other hand, factors specific to Chinese culture, such as shame, may only provoke more social anxiety in early adulthood or in an older population (Zhong et al., 2008; Lau et al., 2009), with no additional effect in social anxiety in younger groups.

It might be true that Chinese CAYA experience a considerable amount of SAS, but this does not necessarily imply that they have a corresponding rate of SAD as their western counterparts did. A criterion of SAD is impairment in social functioning (American Psychiatric Association., 2013). Several cultural factors might reduce the possibility of social dysfunction in the Chinese population. First, in many individualistic cultures, fear and avoidance of social situations are regarded as problematic and might impair social functioning. However, collectivist cultures have greater acceptance of socially reticent behavior (Schreier et al., 2010). Studies have found that in Canadian children, reticent behavior was associated with peer rejection such as overt refusal and disagreement, but the same behavior was associated with positive responses such as approval in a Chinese sample (Chen et al., 2006). In addition, life interference was found to have a stronger relationship with social anxiety in Western youths than in East Asia (Rapee et al., 2011). Second, interpersonal harmony is highly valued in East Asian countries, especially in China (Zhang et al., 2005; Wei and Li, 2013). People might even maintain interpersonal harmony by suppressing their negative emotions (Wei et al., 2013). It is likely for individuals in western cultures to “express appropriate disagreement or disapproval to people they don't know very well” as listed in the LSAS scale (Fresco et al., 2001), whereas Chinese youth might display more fear and avoidance, which reduce the occurrence of social conflicts or dysfunction. Some studies have provided indirect support for this argument. Although Chinese youths feel relatively high levels of anxiety in social situations, they tend not to undermine social norms and interpersonal harmony. It is therefore less likely for them to experience social dysfunction, which is a necessary diagnostic criterion for SAD. However, the cultural factors need to be interpreted with caution. For example, interpersonal harmony may also put more social stress on the individual. In order to maintain interpersonal harmony, individuals may need to compromise and be more attentive to the impact of their behavior on others, which may in turn be a risk factor for social anxiety. More cross-cultural studies may need to be done in the future to explore the reasons for the manifestation of SAS and SAD in China.

The study showed that the prevalence of social anxiety disorder in older CAYA (i.e., aged 15–25) was significantly higher than that of younger CAYA (i.e., 6–15), whereas there was no difference in the prevalence of social anxiety symptoms between the two groups. These finding about the SAD aligns with the existing literature, which suggests that prevalence of SAD is still at a relatively low level in childhood and has a significant increase after early adolescence (Kim et al., 2010; Burstein et al., 2011; Spence et al., 2018). In contrast, the prevalence of SAS remains stable or even decrease from early adolescence to late adolescence (Inderbitzen-Nolan and Walters, 2000; Ranta et al., 2009, 2012). Rapee and Spence (2004) provided a possible explanation that the transition from high social anxiety symptoms to social anxiety disorder depends on the age at which the individual encounters social impairment. From childhood to young adulthood, their perceived social anxiety distress remains unchanged or even decreases, but their social functioning impairment increases when they enter the adolescence. Adolescents usually have to be faced with changes in school environments, physical development, and peer relationships (Simmons, 2017). They might confront more challenges in social aspects, tend to experience impairment in social functioning and therefore are more likely to suffer from SAD.

Although the study showed that the prevalence of SAS and SAD were slightly higher in girls than boys (SAS: 20.7% vs. 18.9; SAD: 5 vs. 4.6%), the differences were not significant (*p* >0.05). This result was in contrast with most of the existing literature, which suggests that females have a higher prevalence of social anxiety than males worldwide and that gender differences are larger in adolescents (Caballo et al., 2014; Asher et al., 2017; Asher and Aderka, 2018). Self-construal, again, might be one reason that explains the non-significant gender differences in the Chinese population. In western culture, men tend to construct and maintain an independent self-construal, whereas women tend to construct and keep an interdependent self-construal (Cross and Madson, 1997). Women's sense of self being more dependent on relationships with others could make them more prone to social anxiety. However, as mentioned above, all individuals in East Asian culture generally have a higher interdependent social construal (Krieg and Xu, 2018). This could make the gender difference in social anxiety among Chinese CAYA relatively small or non-significant. However, the lack of significant gender differences may be due to the broad age range of the study. Research showed that gender differences in social anxiety were more pronounced in mid- and late-adolescence than in childhood and early adolescence (Beesdo et al., 2009). As age increases into adulthood, the gender differences gradually decline (Espinosa et al., 2008). Therefore, combining data from these different age groups may have resulted in an insignificant gender difference.

It is worth noting that there are significant differences in the prevalence estimates between different self-report scales as well as diagnostic tools. For example, the pooled prevalence of SAS assessed by LSAS-SR is significantly higher than SASC, and the prevalence of SAD diagnosed by SCID is significantly higher than MINI-KID. The difference in diagnostic algorithms and the stringency with which these criteria are applied between measurement tools might be one possible factor that leads to differences in the pooled prevalence estimates (Pélissolo et al., 2000).

Strengths of the current meta-analysis include performing a thorough literature search in both English and Chinese databases and estimating prevalence for both SAS and SAD. However, the study has several limitations. First, there is high heterogeneity between studies that has not yet been explained by the hypothesized moderators (e.g., gender, sampling methods, measures). Second, relative to the large population of China, the numbers of studies and participants included in this meta-analysis are still inadequate and underrepresented. The results of this study could provide a reference for researchers and practitioners and cannot replace national, large-scale epidemiological surveys. Third, there was significant heterogeneity across the included studies. One important reason is that different studies used different measurement tools, that is, the prevalence estimates of SAS and SAD assessed by different self-report scales and diagnostic tools significantly differ. Other possible sources of heterogeneity may include differences in the geographic location of the sample, age, and sex ratio. Fourth, the present study included a wide age range (e.g., 6–25). There may be considerable heterogeneity in the prevalence of social anxiety across age groups and a combination of them may be misleading. In addition, studies on children (mean age between 6 and 10 years old) are very few in this meta-analysis, so the findings may be problematic when generalizing to this population. In sum, we need to be cautious when interpreting and applying these results.

## Conclusions and future directions

Overall, our findings revealed that Chinese CAYA frequently experience SAS and SAD. It is hoped that more large-scale epidemiological surveys that use consistent screening and diagnostic tools will be conducted in the future to identify the accurate prevalence of social anxiety in this population. In addition, more culturally sensitive screening and diagnostic tools might need to be developed to identify SAS and SAD. If SAS constantly lead to psychological distress in the Chinese population, the criteria of SAD might need further revision. Finally, prevention and intervention programs to reduce social anxiety in Chinese CAYA are still scarce, and more rigorous randomized controlled trials are needed in the future.

## Data availability statement

The original contributions presented in the study are included in the article/Supplementary material, further inquiries can be directed to the corresponding author.

## Author contributions

XT and ST contributed to the conception and design of the meta-analysis, contributed to the manuscript writing, revision, and approved the submitted version. QL, FC, HT, and XS conducted the coding of studies. All authors listed have made a substantial, direct, and intellectual contribution to the work, and approved it for publication.

## Funding

This work was supported by the Renmin University of China New Faculty Start-Up Grant [22XNKJ27], the Guangdong Planning Office of Philosophy and Social Science [Grant Number GD20YSH06], and the Shenzhen University Natural Science Research Grant [Grant Number 860-000002110172].

## Conflict of interest

The authors declare that the research was conducted in the absence of any commercial or financial relationships that could be construed as a potential conflict of interest.

## Publisher's note

All claims expressed in this article are solely those of the authors and do not necessarily represent those of their affiliated organizations, or those of the publisher, the editors and the reviewers. Any product that may be evaluated in this article, or claim that may be made by its manufacturer, is not guaranteed or endorsed by the publisher.
