# Supplementary material for: Prevalence of social anxiety disorder and symptoms among Chinese children, adolescents and young adults: A systematic review and meta-analysis
Source: Front Psychol. 2022 Aug 22;13:792356. doi: 10.3389/fpsyg.2022.792356 (PMC9442033; doi:10.3389/fpsyg.2022.792356)
Supplement: Supplementary file 1 [file Data_Sheet_1.docx]

**Supplementary Materials**

**Supplementary Material 1: Search strategy**

We applied broad search terms, with an example in Web of Science as follows:

TOPIC: (“social anxiety” or “social phobia” or “socially anxious") AND TOPIC:(minors OR boy* OR girl* OR kid* OR child* OR schoolchild* OR adolescen* OR juvenil* OR youth* OR teen* OR preteen* OR underage* OR pubescen* OR paediatric* OR pediatric* OR student* OR college OR universit*)

Example of Chinese search terms in China National Knowledge Infrastructure Database (CNKI)

SU=('社交焦虑'+‘社交恐惧’+‘社交恐怖’) and SU=('青少年'+'儿童'+'青年'+'男孩'+'女孩'+'高中'+'初中'+'中学'+'初一'+'初二'+'初三'+'高一'+'高二'+'高三'+'大学'+'本科'+'专科'+'大专'+'学生'+'大一'+'大二'+'大三'+'大四')

**Supplementary Material 2: outcome measures**

***Social anxiety disorder***

DAWBA: The Development and Well-Being Assessment

K-SADS: The Kiddie Schedule for Affective Disorders and Schizophrenia

K-SADS-PL: The Schedule for Affective Disorders and Schizophrenia for School-Age Children-Present and Lifetime version

SCID: Structured Clinical Interview for DSM Disorders

SCID-I/P: The SCID Patient Edition

MINI-KID: The Mini-International Neuropsychiatric Interview for Children and Adolescents

CCMD-3: The Chinese Classification of Mental Disorders Version 3

CIDI 3.0: The Composite International Diagnostic Interview Version 3.0

DSM-IV: Diagnostic and Statistical Manual of Mental Disorders (Fourth Edition)

***Social anxiety symptoms***

MASC: Multidimensional Anxiety Scale for Children

SCARED: Revised Screen for Child Anxiety Related Emotional Disorders

SCAS: Spence Children’s Anxiety Scale

SPAI-C: Social Phobia and Anxiety Inventory for Children

SPAI-C-11: Social Phobia and Anxiety Inventory for Children-11-item version

SPAI-B: Brief Form of the Social Phobia and Anxiety Inventory

SASC-R: Social Anxiety Scale for Children-Revised

SAS-A: Social Anxiety Scale for Adolescents

LSAS-CA-SR: Liebowitz Social Anxiety Scale for Children and Adolescents-Self-Report

SPIN: Social Phobia Inventory

**Supplementary Material 3: Risk of Bias Tool**

*External validity (maximum score=4)*

1 Was the study’s target population a close representation of the

national population in relation to relevant variables? (Yes: low risk=1 point; no: high risk=0 points)

2 Was the sampling frame a true or close representation of the target population? (Yes: low risk=1 point; no: high risk=0 points)

3 Was some form of random selection used to select the sample, OR was a census undertaken? (Yes: low risk=1 point; no: high risk=0 points)

4 Was the likelihood of nonresponse bias minimal? (Yes: low risk=1 point; no: high risk=0 points)

*Internal validity (maximum score=6)*

1 Were data collected directly from the subjects (as opposed to a proxy)? (Yes: low risk=1 point; no: high risk=0 points)

2 Was an acceptable case definition used in the study? (Yes: low risk=1 point; no: high risk=0 points)

3 Was the study instrument that measured the parameter of interest shown to have reliability and validity? (Yes: low risk=1 point; no: high risk=0 points)

4 Was the same mode of data collection used for all subjects? (Yes: low risk=1 point; no: high risk=0 points)

5 Was the length of the shortest prevalence period for the parameter of interest appropriate? (Yes: low risk=1 point; no: high risk=0 points)

6 Were the numerator(s) and denominator(s) for the parameter of interest appropriate? (Yes: low risk=1 point; no: high risk=0 points)

**Supplementary Material 4: lists of included studies**

Cai, L. (1998). Survey of Social Situations in 1030 Elementary School Students. *Chinese Journal of School Doctor, 12*(1), 32-33.

Chen, F., Li, H., Ren, X., Su, S., Xin, Z., & Wang, Y. (2016). A 2-year follow-up investigation of social anxiety and its maternal influencing factors of peasant- worker's children in Harbin. *Chinese Journal of Behavioral Medical and Brain Science, 25*(5), 467-471.

Cheng, S. H., Sun, Z. J., Lee, I. H., Lee, C. T., Chen, K. C., Tsai, C. H., . . . Yang, Y. C. (2017). Factors related to self‐reported social anxiety symptoms among incoming university students. *Early intervention in psychiatry, 11*(4), 314-321.

Du, Q., Li, S., Dong, Z., Qiu, C., Liu, K., Meng, Y., & Zhang, W. (2010). The analysis of social anxiety disorder prevalence rates and influencing factors of Chengdu primary school students *Chinese Journal of Behavioral Medicine and Brain Science, 19*(11), 964-966.

Gao, P., Shang, J., He, L., Ren, X., & Wang, Y. (2013). Survery of social anxiety and related influence factors of 3-5 grades studentgs in China. *Chinese Journal of Child Health Care, 21*(1), 90-92.

Guan, B., Luo, X., Deng, Y., Wei, Z., Ye, H., Yuan, X., . . . Ding, J. (2010). Prevalence of psychiatric disorders in primary and middle school students in Hunan Province. *Chinese Journal of Contemporary Pediatrics, 12*(2), 123-127.

Li, J., Liu, Y., Wan, H., & Mei, S. (2011). Correlation between social anxiety and psychological traits among university students. *Chinese Journal of Public Health, 27*(9), 1073-1075.

Li, M., Tang, G., Zhou, L., Yu, Z., & Mao, H. (2015). Association between social anxiety and excessive daytime sleepiness among college students. *Chinese Journal of School Health, 36*(11), 1675-1677.

Lin, S., Wang, D., Xian, Y., Yang, J., & Wu, P. (2018). Current status of social anxiety and depression among primary school students in Urumqi, China. *Chinese Journal of Contemporary Pediatrics, 20*(8), 670-674.

Luan, Y., Cao, J., Zhou, Y., Yang, J., & Yang, J. (2014). The prevalence rate and growth environmental factors of social anxiety disorder among Daqing college students. *Chinese Journal of Behavioral Medicine and Brain Science, 23*(9), 793-796.

Qiu, W., & Kang, K. (2014). Social anxiety of rural children in the fourth grade of a primary school. *Journal of Campus Life and Mental Health, 12*(4), 247-248.

Qu, Y., Jiang, H. Y., Zhang, N., Wang, D. H., & Guo, L. T. (2015). Prevalence of Mental Disorders in 6-16-Year-Old Students in Sichuan Province, China. *International Journal of Environmental Research and Public Health, 12*(5), 5090-5107. doi:10.3390/ijerph120505090

Shang, J., Chen, X., He, L., Ren, X., & Wang, Y. (2012). Effect of maternal anxiety, depression, acceptance and psychological control on students’ social anxiety. *Chinese Journal of Behavioral Medical and Brain Science, 21*(9), 778-780.

Su, C., Huang, G., Hu, L., Zhang, X., Ni, S., Lin, J., & Zhou, X. (2006). Epidemiological Survey of Social Anxiety Disorder in Pupils of Zhanjiang Urban Districts. *Chinese Mental Health Journal, 20*(11), 736-738.

Su, L., Yin, Q., Wang, K., Zhu, Y., Tang, J., & Luo, X. (2003). Anxiety disorders in Chinese elementary school pupils. *Chinese Journal of Nervous and Mental Diseases, 29*(5), 330-333.

Su, S., Ren, X., Chen, F., Li, H., & Wang, Y. (2015). Study on 4-year follow-up of the students’ social anxiety detection about 684 students in Harbin. *Chinese Journal of Child Health Care, 23*(5), 465-467.

Wang, H., Zhang, R., Chen, Y., Wang, H., Zhang, Y., Gan, J., . . . Tan, Q. (2014). Social anxiety disorder in the Chinese military: prevalence, comorbidities, impairment, and treatment-seeking. *Psychiatry Research, 220*(3), 903-908.

Wang, J. (2010). Relationship between social anxiety and type of temperament in college students. *Chinese Journal of School Doctor, 24*(7), 500-501.

Wang, L., Zhang, S., Sun, Y., & Zhang, X. (2006). Social Anxiety and the Influencing Factors Among Pupils in Rural Area in Anhui. *Chinese Journal of School Health, 27*(10), 853-855.

Wei, Z., & Huo, L. (2009). Study on the Relationship Between Social Anxiety and Adult Attachment Styles of College Students. *China Journal of Health Psychology, 17*(8), 954-957.

Wei, Z., Sun, H., & Huo, L. (2011). Study on the relationship between social anxiety and adult attachment styles among junior college nursing students. *Journal of Nursing Administration, 11*(8), 554-556.

Wu, Y.-L., Zhao, X., Li, Y.-F., Ding, X.-X., Yang, H.-Y., Bi, P., & Sun, Y.-H. (2016). The risk and protective factors in the development of childhood social anxiety symptoms among Chinese children. *Psychiatry Research, 240*, 103-109.

Xiao, R., Wu, W., Hu, J., Qiu, C., Qiang, W., Geng, W., . . . Zhang, W. (2006). Prevalence and Risk Factors of Social Anxiety Disorder in High Schools and Universities in Chengdu. *Journal of Sichuan University (Medical Science Edition), 37*(4), 636-640.

Yang, H., & Xu, H. (2003). A survery and treatment of primary school students' social anxiety. *Journal of Chinese Society of Education, 2*, 46-47.

Ye, H., Luo, X., Yuan, X., Wei, Z., Guan, B., & Ning, Z. (2013). Epidemiological investigatiion of anxiety disorders in primary and middle school students in Changsha area. *Journal of Xinxiang Medical University, 30*(10), 814-816.

Yu, X., Zhu, X., Zheng, H., Zhao, Y., Ma, H., & Cheng, S. (2015). Relationship of parents' upbringing mode, type of temperament and children's social anxiety. *Journal of Hebei United University (Health Sciences), 17*(4), 46-49.

Zhang, J., Zhao, B., Yao, H., Sun, X., & Zhang, C. (2018). Prevalence of mental disorders in 6-16-year-old students in Zhenjiang, Jiangsu Province. *Journal of Neuroscience and Mental Health, 18*(10), 718-722.

Zhang, Q., & Liu, C. (2011). An Epidemiological Survey of Social Anxiety Disorders among College Students in Qingzhou. *Journal of China Traditional Chinese Medicine Information, 3*(3), 43.

**Supplementary Material 5: Pooled Prevalence of Social Anxiety Disorder**

**Supplementary Material 6: Pooled Prevalence of Social Anxiety Symptoms**

**Supplementary Material 7: Funnel Plot for Social Anxiety Disorder**

**Supplementary Material 8: Funnel Plot for Social Anxiety Symptoms**
